# HIV-1 Infection Alters Post-Transcriptional Processing of HTLV-1 RNA in MT-2 Cells

**DOI:** 10.3390/microorganisms14102359

**Published:** 2026-10-15

**Authors:** Haoting Liu, Shuanglong Wei, Jingwan Han, Hanping Li, Yongjian Liu, Lei Jia, Xiaolin Wang, Bohan Zhang, Lin Li

**Affiliations:** 1 School of Basic Medical Sciences, Anhui Medical University, Hefei 230032, China; l2393448454@126.com; 2 State Key Laboratory of Pathogen and Biosecurity, Academy of Military Medical Sciences, Beijing 100071, China; a384305818@163.com (S.W.); hanjingwan@outlook.com (J.H.); yongjian325@sina.com (Y.L.); 15001193408@163.com (L.J.); woodsxl@163.com (X.W.); 3 School of Public Health and Health Management, Gannan Medical University, Ganzhou 341000, China

**Keywords:** HTLV-1, HIV-1, direct RNA sequencing, alternative splicing, RNA modifications

## Abstract

Human T-lymphotropic virus type 1 (HTLV-1) gene expression is extensively regulated at the post-transcriptional level, but how human immunodeficiency virus type 1 (HIV-1) infection affects the processing of HTLV-1 RNA remains unclear. Here, we combined reverse transcription quantitative PCR (RT-qPCR) with Oxford Nanopore direct RNA sequencing (ONT DRS) to characterize HTLV-1 RNA levels, alternative splicing, poly(A)-tail length, and RNA modifications in HIV-1-infected MT-2 cells. HIV-1 infection did not significantly alter total HTLV-1 RNA levels but was associated with changes in HTLV-1 RNA processing. D1-A8 splicing became predominant, and the proportion of p21^rex^ transcripts increased from 29.45% to 82.07% of total HTLV-1 transcripts. Poly(A)-tail changes were transcript-specific, with longer tails in env and shorter tails in p21^rex^, HBZ, and unannotated transcripts. HIV-1 infection also altered N^6^-methyladenosine (m^6^A), 5-methylcytidine (m^5^C), and pseudouridine (Ψ) modifications, with more extensive changes in m^5^C and Ψ than in m^6^A. Demethylation mediated by alpha-ketoglutarate-dependent dioxygenase AlkB homolog 5 (ALKBH5) further supported the assignment of nucleotides 7011, 7357, and 7691 as m^6^A modification sites. Together, these findings demonstrate that HIV-1 infection alters the post-transcriptional regulation of HTLV-1 RNA despite stable total HTLV-1 RNA levels.

## 1. Introduction

Viral coinfection can influence viral replication, gene expression, and disease progression through direct virus–virus interactions or alterations in the host cellular environment. Such interactions have been described in coinfections involving SARS-CoV-2 and influenza virus, as well as human immunodeficiency virus type 1 (HIV-1) and mpox virus [1,2,3,4,5,6]. Retroviral interactions are particularly relevant because different retroviruses may depend on overlapping host pathways for transcription, RNA processing, and nuclear export.

Human T-lymphotropic virus type 1 (HTLV-1), the first human retrovirus to be identified, establishes persistent infection through reverse transcription of its RNA genome followed by integration of proviral DNA into the host genome [7,8]. HIV-1 follows a similar retroviral replication strategy and likewise depends extensively on host cellular machinery for viral gene expression. Thus, when HIV-1 infects HTLV-1-positive cells, the two retroviruses coexist within the same cellular environment and may influence not only viral transcription but also the subsequent processing of viral RNAs.

HIV-1/HTLV-1 coinfection has been recognized since the 1980s. Previous studies showed that HTLV-1/2 tax/rex transcripts and viral antigens were detected more frequently in individuals coinfected with HIV-1 [9]. Roy et al. further reported that exposure to HIV-1 increased the proportion of HTLV-1 antigen-positive cells, and that recombinant HIV-1 Tat enhanced HTLV-1 p19 production under experimental conditions [10]. These findings demonstrate that HIV-1 can influence HTLV-1 expression [11,12]. However, previous studies have largely focused on viral antigens, selected transcripts, or overall RNA levels, and whether HIV-1 alters the processing and modification of HTLV-1 transcripts remains unclear.

RNA processing is a major determinant of HTLV-1 gene expression and viral protein production [13,14,15,16,17,18,19,20]. Alternative splicing generates unspliced, singly spliced, and multiply spliced RNAs encoding structural, regulatory, and accessory proteins [21,22,23,24,25,26,27], while poly(A)-tail length can influence RNA stability and translation [28,29,30]. RNA modifications, including N^6^-methyladenosine (m^6^A), 5-methylcytidine (m^5^C), and pseudouridine (Ψ), provide an additional layer of post-transcriptional regulation by affecting RNA structure, RNA–protein interactions, processing, and translation [31,32,33,34,35,36,37,38]. Together, these processes shape the composition and molecular properties of HTLV-1 transcripts and therefore represent important levels of viral gene regulation.

In the present study, we used MT-2 cells harboring multiple integrated HTLV-1 proviruses as a model to investigate how HIV-1 infection affects the post-transcriptional processing of HTLV-1 RNA [39,40,41,42,43]. We combined reverse transcription quantitative PCR (RT-qPCR) with Oxford Nanopore direct RNA sequencing (ONT DRS) to characterize changes in HTLV-1 RNA levels, splice-site usage, transcript composition, poly(A)-tail length, and m^6^A, m^5^C, and Ψ modifications following HIV-1 infection. By resolving these changes at the transcript level, we sought to define how HIV-1 influences HTLV-1 RNA processing and post-transcriptional regulation.

## 2. Materials and Methods

### 2.1. MT-2 Cell Culture

MT-2 cells (RRID: CVCL_2631) were obtained from the American Type Culture Collection (ATCC, Manassas, VA, USA) and cultured in RPMI 1640 medium (Gibco, Thermo Fisher Scientific, Waltham, MA, USA) supplemented with 10% fetal bovine serum, 100 U/mL penicillin, and 100 μg/mL streptomycin at 37 °C in a humidified atmosphere containing 5% CO_2_. Cells were passaged every 2 days to maintain exponential growth. After at least two passages following recovery from cryopreservation, cultures with a density of ≥1 × 10^6^ cells/mL and viability of ≥98% were used for subsequent experiments.

### 2.2. HIV-1 Infection of MT-2 Cells

MT-2 cell density and viability were determined by trypan blue exclusion using an automated cell counter. The infectious titer of HIV-1 NL4-3 was determined in TZM-bl cells (RRID: CVCL_B478) using a 50% tissue culture infectious dose (TCID_50_) assay and expressed as TCID_50_/mL. Based on the measured TCID_50_ titer, the viral inoculum was calculated such that 5 × 10^5^ TCID_50_ were applied to 1 × 10^6^ MT-2 cells, corresponding to a nominal multiplicity of infection (MOI) of 0.5. A total of 1 × 10^6^ viable MT-2 cells were seeded into one well of a six-well plate and infected with the calculated amount of HIV-1 NL4-3 in complete RPMI 1640 medium (final volume, 5 mL). At 36 h post-infection, cells were collected by centrifugation at 300× *g* for 5 min at room temperature, and the resulting cell pellet was collected for subsequent experiments.

### 2.3. Total RNA Extraction and Quality Assessment

Total RNA was extracted from MT-2 cells and HIV-1-infected MT-2 cells using the TaKaRa MiniBEST Universal RNA Extraction Kit (Takara Bio, Kusatsu, Japan; 9767) according to the manufacturer’s instructions. Purified RNA was eluted in 50 μL of RNase-free water. RNA concentration and purity were assessed using a NanoDrop™ One spectrophotometer (Thermo Fisher Scientific, Waltham, MA, USA). Samples with RNA concentrations ≥ 125 ng/μL, A260/A280 ratios of 1.8–2.0, and A260/A230 ratios of 1.8–2.0 were used for subsequent experiments.

### 2.4. Quantification of Viral RNA Levels

HIV-1 RNA in total RNA extracted from HIV-1-infected MT-2 cells was quantified using an HIV-1 RNA Quantitative Diagnostic Kit (PCR-Fluorescence Probing; Daan Gene Co., Ltd., Guangzhou, China) according to the manufacturer’s instructions.

HTLV-1 RNA was quantified using the HTLV-1 Viral Load Assay Kit (Tiandz, Beijing, China; 15-87810). The supplied standard was subjected to serial 10-fold dilutions to prepare six concentration standards, labeled A-F from highest to lowest. Total RNA extracted from control and HIV-1-infected MT-2 cells was diluted 20-fold before analysis. Each 20-μL reaction contained 10 μL of reaction buffer, 1 μL of enzyme mixture, 3 μL of primer–probe mixture, and 6 μL of standard or diluted RNA sample. All standards and samples were analyzed in duplicate using a LightCycler^®^ 480 Real-Time PCR System (Roche Diagnostics, Rotkreuz, Switzerland). Reverse transcription was performed at 50 °C for 10 min, followed by initial denaturation at 95 °C for 10 min and 45 cycles of 95 °C for 15 s and 60 °C for 60 s, with fluorescence acquired at 60 °C. A standard curve was generated using the six standards, and HTLV-1 RNA levels were determined by interpolation of the sample Ct values against the standard curve.

### 2.5. In Vitro Transcription

To generate unmodified RNA controls for position-specific background correction in the subsequent RNA modification analysis, six primer pairs targeting selected regions of the HTLV-1 genome were designed using NCBI Primer-BLAST (Supplementary Table S1). The T7 promoter sequence was appended to the 5′ end of each forward primer to generate double-stranded DNA templates for in vitro transcription.

DNA templates were amplified from total RNA extracted from MT-2 cells using the PrimeScript™ One Step RT-PCR Kit Ver.2 (Takara Bio, Shiga, Japan; RR055A). Each 50-μL reaction contained up to 1 μg of total RNA and 0.4 μM of each primer. Reverse transcription was performed at 50 °C for 30 min, followed by initial denaturation at 94 °C for 2 min and 35 cycles of 94 °C for 15 s, 52 °C for 20 s, and 72 °C for 100 s.

Amplicons of the expected sizes were purified from agarose gels using the Wizard^®^ SV Gel and PCR Clean-Up System (Promega, Madison, WI, USA; A9282). In vitro transcription was performed with 0.1–0.5 μg of purified DNA template using the T7 High Yield RNA Transcription Kit (Vazyme, Nanjing, China; TR101) at 37 °C for 2 h. Residual DNA templates were removed by DNase I digestion at 37 °C for 15 min. The resulting RNA transcripts were purified using the Monarch^®^ Spin RNA Cleanup Kit (New England Biolabs, Ipswich, MA, USA; T2050L) and eluted in 50 μL of nuclease-free water.

### 2.6. Nanopore Direct RNA Sequencing

The control MT-2 direct RNA sequencing datasets used for comparison in the present study were previously generated and reported by Wei et al. [44]. These control datasets were generated using the same ONT DRS platform and kit under comparable library preparation and sequencing conditions.

Nanopore direct RNA sequencing libraries were prepared from 1 μg of total RNA using the Direct RNA Sequencing Kit (SQK-RNA004; Oxford Nanopore Technologies, Oxford, UK) according to the manufacturer’s protocol, with minor modifications [45,46,47,48]. Briefly, 1 μg of total RNA in 8 μL was ligated to the RT Adapter in a 15-μL reaction containing 3 μL of NEBNext Quick Ligation Reaction Buffer (New England Biolabs, Ipswich, MA, USA; B6058), 1 μL of RNaseOUT™ Recombinant Ribonuclease Inhibitor, 1 μL of RT Adapter, 1.5 μL of T4 DNA Ligase (New England Biolabs; M0202), and 0.5 μL of nuclease-free water. The reaction was incubated at room temperature for 20 min.

First-strand cDNA synthesis was performed using SuperScript™ III Reverse Transcriptase (Thermo Fisher Scientific, Waltham, MA, USA; 18080044) at 50 °C for 50 min, followed by enzyme inactivation at 70 °C for 10 min. The resulting RNA-cDNA hybrids were purified using Agencourt RNAClean XP beads (Beckman Coulter, Brea, CA, USA; A63987) and eluted in 20 μL of nuclease-free water.

The sequencing adapter was then ligated to the purified RNA-cDNA hybrids with RNA Ligation Adapter, NEBNext Quick Ligation Reaction Buffer, and T4 DNA Ligase for 20 min at room temperature. The adapted libraries were purified using RNAClean XP beads, eluted in 13 μL of RNA Elution Buffer, and quantified using the Qubit™ dsDNA HS Assay Kit (Thermo Fisher Scientific; Q32851).

RNA flow cells (FLO-MIN004RA; Oxford Nanopore Technologies) were primed according to the manufacturer’s instructions. For each sequencing run, 12 μL of the prepared library was mixed with 37.5 μL of Sequencing Buffer and 25.5 μL of Library Solution and loaded onto the flow cell through the SpotON sample port. Direct RNA sequencing was performed for 72 h using an Oxford Nanopore Technologies MinION Mk1C sequencing device controlled by MinKNOW software (version 24.06.15).

### 2.7. Bioinformatics Analysis

For comparative analysis, the previously published control MT-2 datasets [44] and the HIV-1-infected MT-2 datasets were processed using the same bioinformatics workflow described below.

Raw nanopore signals in POD5 format were basecalled using Dorado v0.8.1 with the RNA004 super-accuracy model (rna004_130bps_sup@v5.1.0). Models for m^6^A within DRACH motifs (m6A_DRACH), m^5^C, and Ψ were enabled during basecalling [49,50,51,52]. Poly(A)-tail lengths were estimated using the --estimate-poly-a option. Modified-base probabilities and poly(A)-tail estimates were retained in the resulting modBAM files. Reads with mean basecalling Q scores < 10 were removed using SAMtools v1.2.1 [53].

For splice-junction analysis, the quality-filtered Dorado BAM files were converted to FASTQ format using SAMtools. The resulting reads were independently aligned to the HTLV-1 reference genome (GenBank accession no. AF033817.1) using minimap2 v2.28 [54] with the -x splice, -uf, and -k14 options. The alignments were coordinate-sorted and indexed using SAMtools. Sequencing yield, read-length distribution, and mean read-quality distribution were assessed using NanoPlot v1.42.0 [55], and alignment statistics were obtained using the flagstat and stats functions of SAMtools. Splice junctions and junction-supporting read counts were extracted from the splice-aware alignments using Megadepth v1.2.0 [56]. Junctions were filtered according to mapping quality, read support, and splice-site sequence. Canonical GT-AG junctions were prioritized, whereas noncanonical junctions were retained only when supported by reference annotations or multiple independent high-quality reads. Reads were assigned to individual isoforms based on their complete ordered splice-junction chains, after which splice-site usage and isoform abundance were quantified [57].

For modified-base analysis, the quality-filtered Dorado modBAM files, which retained the modified-base annotations generated during basecalling, were analyzed using Modkit v0.5.0. Position-level modification ratios and per-read modification probabilities were obtained for m^6^A, m^5^C, and Ψ. At each nucleotide position, the modification ratio was calculated as the number of valid reads with a modification probability > 0.80 divided by the total number of valid reads covering that position, expressed as a percentage. Unmodified in vitro-transcribed RNA covering the corresponding HTLV-1 regions was analyzed in parallel to estimate position-specific background signals. Background correction was performed by directly subtracting the modification ratio of the in vitro-transcribed RNA control from that of each experimental sample at the same nucleotide position. Negative corrected values were set to zero. High-confidence candidate modification sites were defined as positions with a background-corrected modification ratio ≥ 20% and valid coverage ≥ 1000 reads [44,58,59,60,61].

For isoform-specific modification analysis, per-read modification calls from the modBAM files were linked to splice-isoform assignments from the minimap2 alignments using read identifiers. Modification ratios were calculated separately for each isoform and corrected by subtracting the corresponding modification ratios detected in the in vitro-transcribed RNA control.

### 2.8. In Vitro RNA Demethylation

A total of 3 μg of RNA was incubated with 3 μg of recombinant alpha-ketoglutarate-dependent dioxygenase AlkB homolog 5 (ALKBH5) protein (Active Motif, Carlsbad, CA, USA; 31589) in a reaction mixture containing 100 mM KCl, 2 mM MgCl_2_, 0.2 U/μL SUPERase·In™ RNase inhibitor, 2 mM ascorbic acid, 300 μM α-ketoglutarate, 150 μM ammonium iron (II) sulfate hexahydrate, and 50 mM HEPES (pH 6.5) at 37 °C for 2 h. The reaction was terminated by adding 2 μL of 0.5 M EDTA, and the RNA was subsequently purified using the TaKaRa RNA extraction kit as described above.

For the mock-treated control, an equal volume of PBS was added instead of recombinant ALKBH5, while all other reaction components and incubation conditions were kept identical.

### 2.9. Quantification of Global m^6^A Levels

Global m^6^A levels in ALKBH5-treated and mock-treated RNA samples were quantified using the EpiQuik™ m^6^A RNA Methylation Quantification Kit (Colorimetric; EpigenTek, Farmingdale, NY, USA; P-9005) according to the manufacturer’s instructions. Briefly, 200 ng of RNA was immobilized in each assay well, with positive and negative controls included in parallel. Following incubation at 37 °C for 90 min, the wells were sequentially incubated with the capture antibody, detection antibody, and enhancer solution. Color was developed using the developer solution and terminated when the positive control reached the recommended intensity. Absorbance was measured at 450 nm using a microplate reader.

### 2.10. Statistical Analysis

Statistical analyses were performed using GraphPad Prism 10.1.2. Independent biological replicates were included for each experimental condition. For sequencing-based analyses, individual reads were used to characterize read-level distributions and transcript-specific features and were not considered independent biological replicates. Where applicable, data are presented as mean ± SD from independent experiments. Statistical comparisons between two groups were performed using an unpaired two-tailed Student’s *t*-test. Statistical significance was denoted by * *p* < 0.05, ** *p* < 0.01, *** *p* < 0.001, and **** *p* < 0.0001; ns, *p* ≥ 0.05.

## 3. Results

### 3.1. Viral RNA Levels and Direct RNA Sequencing Quality Assessment

**Figure 1:**
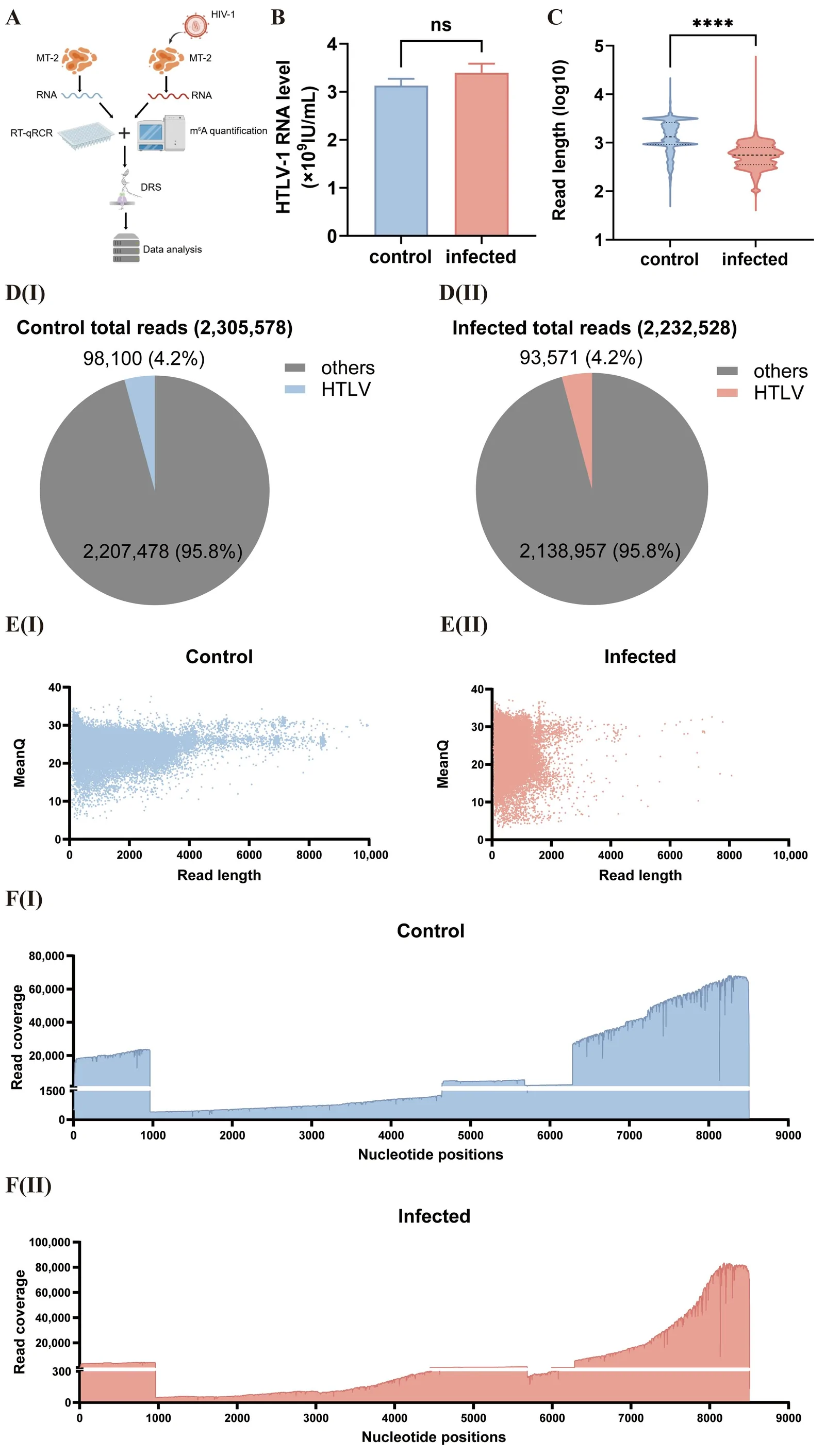
Statistics and characteristics of Nanopore direct RNA sequencing data from control and HIV-1-infected MT-2 cells. (**A**) Experimental workflow for sample preparation, RNA extraction, RT-qPCR, m^6^A quantification, Nanopore direct RNA sequencing, and data analysis. (**B**) HTLV-1 RNA levels in control and HIV-1-infected MT-2 cells. (**C**) Log10-transformed length distributions of HTLV-1 reads in the two groups. (**D**) Numbers and proportions of HTLV-1 and non-HTLV-1 reads among the total sequencing reads. (**E**) Scatter plots showing the relationship between read length and mean Q score in each group. (**F**) Read coverage across the HTLV-1 genome in the two groups. The control MT-2 direct RNA sequencing data shown in panels (**C**–**F**) were previously reported by Wei et al. [44] and are presented here for comparison with the HIV-1-infected condition. ns, *p* ≥ 0.05; **** *p* < 0.0001.

Before direct RNA sequencing, total HTLV-1 RNA levels were comparable between control and HIV-1-infected MT-2 cells (Figure 1B), with successful HIV-1 infection confirmed by HIV-1 RNA quantification (Supplementary Figure S1). For the representative comparison, the previously published control MT-2 dataset [44] contained 2,305,578 reads, whereas the HIV-1-infected MT-2 dataset contained 2,232,528 reads. Among these, 98,100 and 93,571 reads mapped to the HTLV-1 reference genome, respectively, accounting for approximately 4.2% of total reads in both datasets (Figure 1D). HTLV-1-mapped reads covered the entire viral genome at a depth exceeding 50× (Figure 1F), and mean Q scores were approximately 25 in both datasets (Figure 1E), indicating sufficient sequencing depth and comparable read quality. Two biological replicates were available for each condition and showed consistent patterns. Representative datasets are shown in the main figures, whereas the second replicate is provided in Supplementary Figure S2. The control MT-2 sequencing datasets for both replicates were previously reported by Wei et al. [44].

Genome-wide coverage profiles were broadly similar between the two datasets, although lower coverage was observed in the 5′ region of the HTLV-1 genome in the HIV-1-infected sample (Figure 1F). The control dataset exhibited a bimodal read-length distribution, with peaks at approximately 1000 and 3500 nucleotides, whereas the HIV-1-infected dataset showed a broader distribution with less distinct peaks (Figure 1C). Thus, despite comparable total HTLV-1 RNA levels, differences in HTLV-1 read-length distribution and 5′-end coverage were observed between the two conditions.

### 3.2. HIV-1 Infection Remodels HTLV-1 Alternative Splicing Profiles

**Figure 2:**
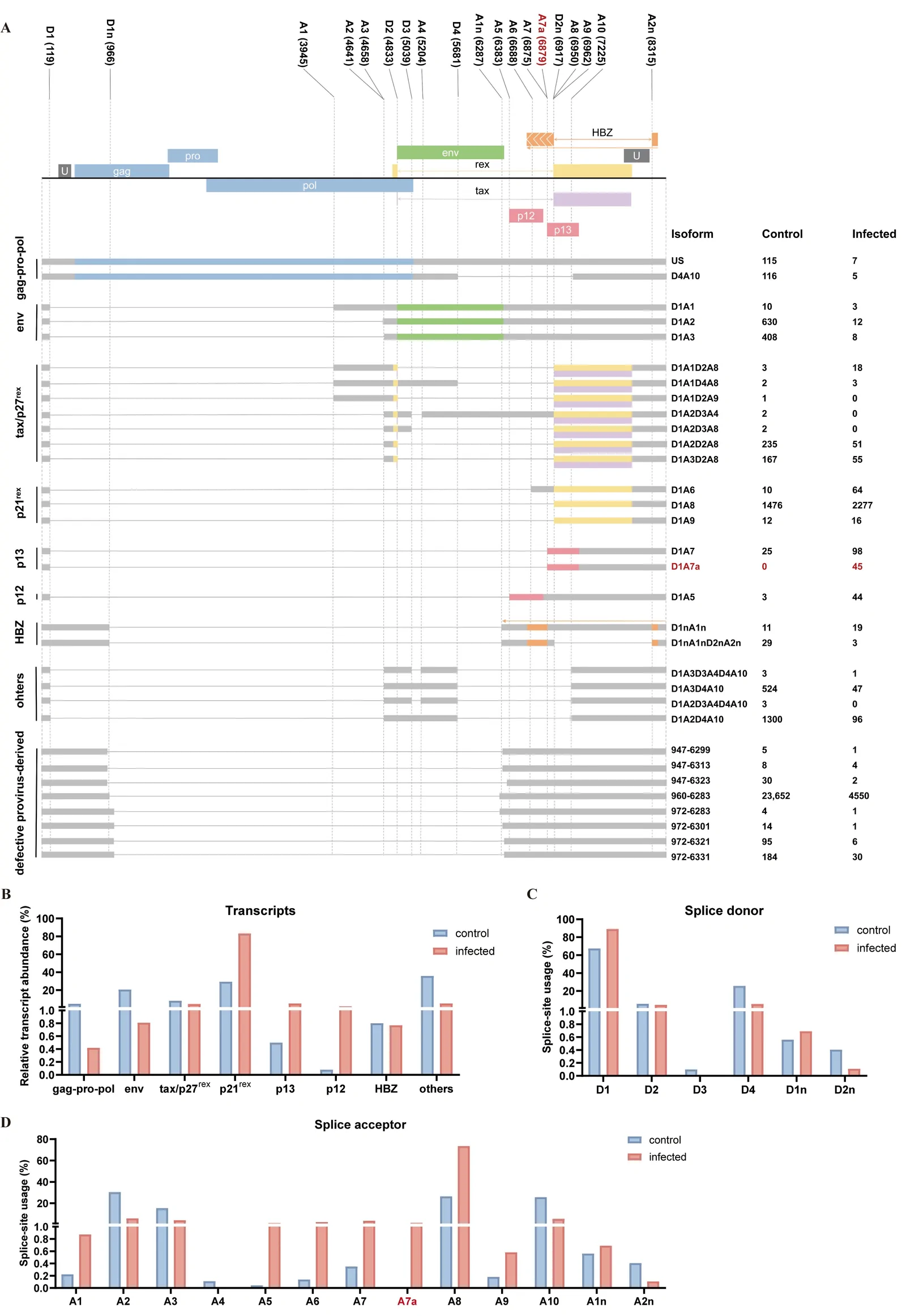
HTLV-1 transcript isoform profiles, relative transcript abundance, and splice-site usage in control and HIV-1-infected MT-2 cells. (**A**) HTLV-1 genome organization, splice sites, and transcript isoforms detected in control and HIV-1-infected MT-2 cells. Read counts for individual isoforms are indicated on the right. The A7a acceptor site and candidate D1-A7a isoform identified in this study are highlighted in red. (**B**) Relative abundances of HTLV-1 transcripts in control and HIV-1-infected MT-2 cells. (**C**) Comparison of HTLV-1 splice donor site usage between control and HIV-1-infected MT-2 cells. (**D**) Comparison of HTLV-1 splice acceptor site usage between control and HIV-1-infected MT-2 cells. The control MT-2 direct RNA sequencing data used for the transcript isoform and splice-site analyses were previously reported by Wei et al. [44] and are presented here for comparison with the HIV-1-infected condition.

In the previously published control MT-2 dataset [44], twenty-three HTLV-1 RNA isoforms were identified and assigned to eight transcript categories (Figure 2A). The tax/p27^rex^ category showed the greatest isoform diversity, comprising seven splice variants. However, the most abundant transcript categories were unannotated isoforms classified as “others” and p21^rex^, which accounted for 35.97% and 29.45% of the total HTLV-1 transcript population, respectively. All unannotated isoforms used the conserved D1 splice-donor site, which was also the predominant donor site overall. Acceptor-site usage was concentrated at A2, A3, A8, and A10, together representing more than 98% of all detected acceptor events.

The composition of the HTLV-1 transcript population differed between control and HIV-1-infected cells. Twenty isoforms were detected in HIV-1-infected cells, including one previously unannotated isoform, whereas four low-abundance isoforms present in control cells were no longer detected (Figure 2A). The most pronounced change was an increase in the relative abundance of p21^rex^ transcripts from 29.45% to 82.07%. Meanwhile, the proportion of unannotated isoforms decreased from 35.97% to 5.01%. The relative abundance of env and gag-pro-pol transcripts also decreased, whereas p12 and p13 transcripts became more highly represented (Figure 2B).

Splice-site usage changed accordingly. D1 usage increased to 90.38%, while D4 usage declined and D3-associated splicing was not detected in the HIV-1-infected dataset (Figure 2C). Among the acceptor sites, A8 showed the largest increase, rising from 26.00% to 76.56%, consistent with the enrichment of D1-A8-spliced p21^rex^ transcripts. In contrast, usage of A2, A3, and A10 decreased. A previously unannotated acceptor site adjacent to A7 was also detected and designated A7a (Figure 2D); its use generated a candidate novel D1-A7a isoform identified in HIV-1-infected cells (Figure 2A).

### 3.3. HIV-1 Infection Changes HTLV-1 Poly(A)-Tail Length

**Figure 3:**
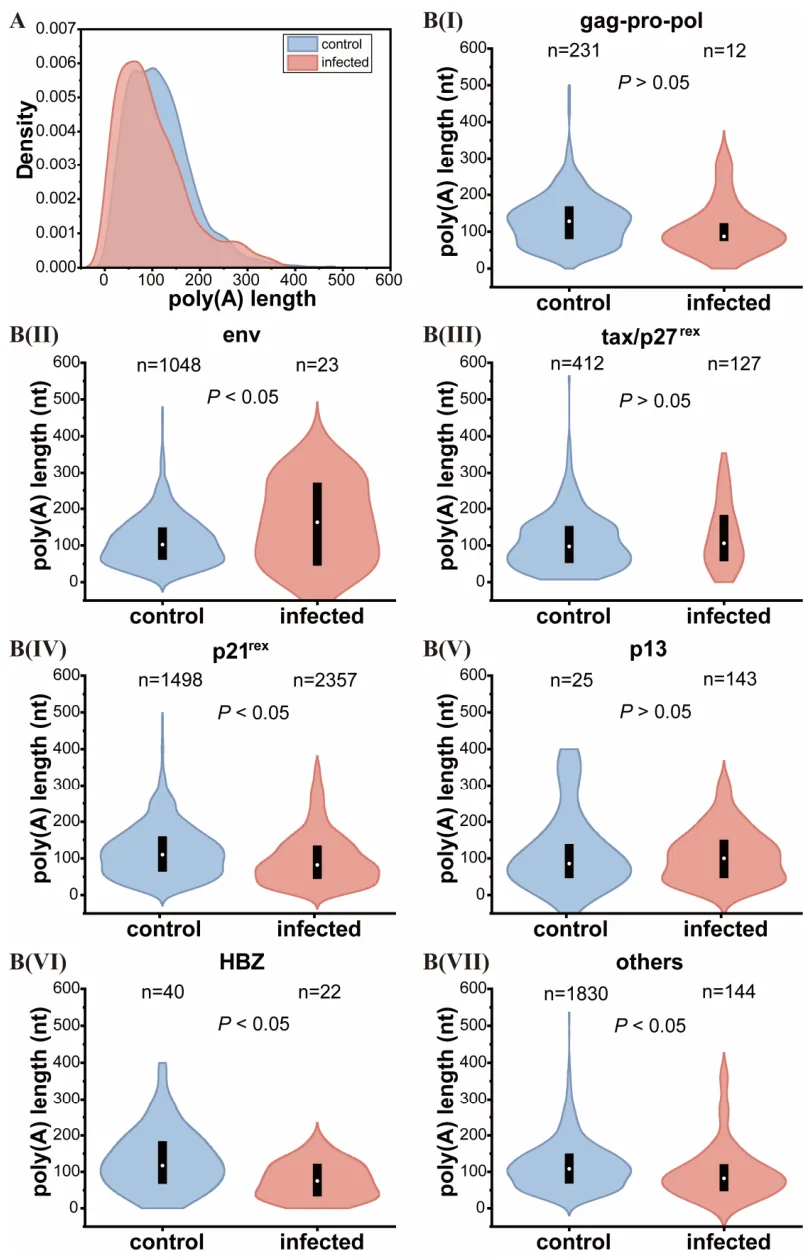
Poly(A)-tail length distributions of HTLV-1 transcripts in control and HIV-1-infected MT-2 cells. (**A**) Overall poly(A)-tail length distributions of HTLV-1 transcripts in control and HIV-1-infected MT-2 cells. (**B**) Poly(A)-tail length distributions of different HTLV-1 transcript classes in the two groups. *n* indicates the number of individual transcript molecules assigned to each transcript class and included in the poly(A)-tail analysis. The control MT-2 direct RNA sequencing data used for the poly(A)-tail analysis were previously reported by Wei et al. [44] and are presented here for comparison with the HIV-1-infected condition.

HIV-1 infection shifted the HTLV-1 poly(A)-tail distribution toward shorter lengths (Figure 3A). In control MT-2 cells, the distribution was right-skewed, with a peak at approximately 120 nt and only a small proportion of transcripts extending beyond 400 nt. In HIV-1-infected cells, the peak shifted to approximately 100 nt, and the distribution became narrower.

Poly(A)-tail lengths were further compared across individual transcript categories (Figure 3B). After HIV-1 infection, env transcripts showed a significant increase in poly(A)-tail length, whereas significant shortening was observed for p21^rex^, HBZ, and unannotated “others” transcripts (*p* < 0.05). No significant differences were detected for gag-pro-pol, tax/p27^rex^, or p13 transcripts (*p* > 0.05). The p12 transcripts were excluded because the number of observations was insufficient for a reliable comparison.

### 3.4. HIV-1 Infection Alters RNA Modification Profiles in HTLV-1 Transcripts

**Figure 4:**
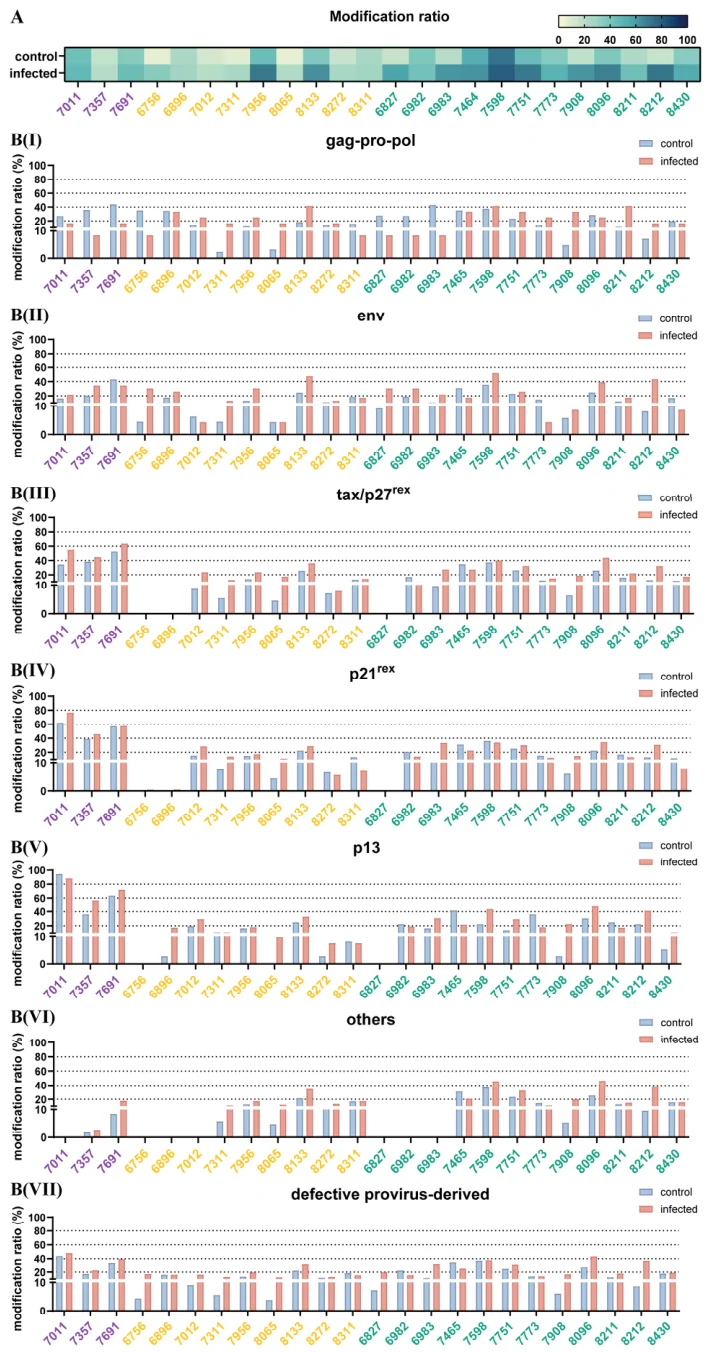
RNA modification profiles of HTLV-1 transcripts in control and HIV-1-infected MT-2 cells. (**A**) Overall distribution of m^6^A, m^5^C, and Ψ modification sites in HTLV-1 transcripts. (**B**) Changes in modification ratios of m^6^A, m^5^C, and Ψ sites across HTLV-1 transcript classes following HIV-1 infection. The colors of the nucleotide position labels on the x-axis indicate modification types: purple, m^6^A; orange, m^5^C; and green, Ψ. The control MT-2 direct RNA sequencing data used for the RNA modification analysis were previously reported by Wei et al. [44] and are presented here for comparison with the HIV-1-infected condition.

We next analyzed RNA modification profiles of HTLV-1 transcripts in control and HIV-1-infected MT-2 cells. Candidate modification sites were identified using predefined thresholds, with unmodified in vitro-transcribed RNA serving as the background reference. Fifteen sites were detected in control cells, including two m^6^A, four m^5^C, and nine Ψ sites. Among these, the Ψ site at nucleotide 7598 showed the highest modification ratio, reaching approximately 72%. Modification ratios at the same genomic positions varied among transcript classes, and the detection of individual sites also depended on transcript structure because alternatively spliced regions were absent from isoforms lacking the corresponding sequences. Following HIV-1 infection, nine additional sites met the detection thresholds, including one m^6^A, five m^5^C, and three Ψ sites. For many sites detected under both conditions, modification ratios were higher in the HIV-1-infected condition (Figure 4A). Overall, changes in m^5^C and Ψ involved more sites and a broader range of modification ratios than those observed for m^6^A.

The extent of these changes differed among HTLV-1 transcript classes (Figure 4B). More pronounced increases in modification ratios were observed in env, tax/p27^rex^, and defective-provirus-derived transcripts, whereas p21^rex^, p13, and unannotated transcripts showed more moderate changes. HBZ transcripts were excluded from transcript-level analysis because of insufficient read counts. In most transcript classes, sites showing increased modification ratios outnumbered those showing decreases, whereas gag-pro-pol transcripts exhibited comparatively limited changes. These results indicate that the effects of HIV-1 infection on HTLV-1 RNA modification were transcript-dependent rather than uniform across the viral transcriptome.

**Figure 5:**
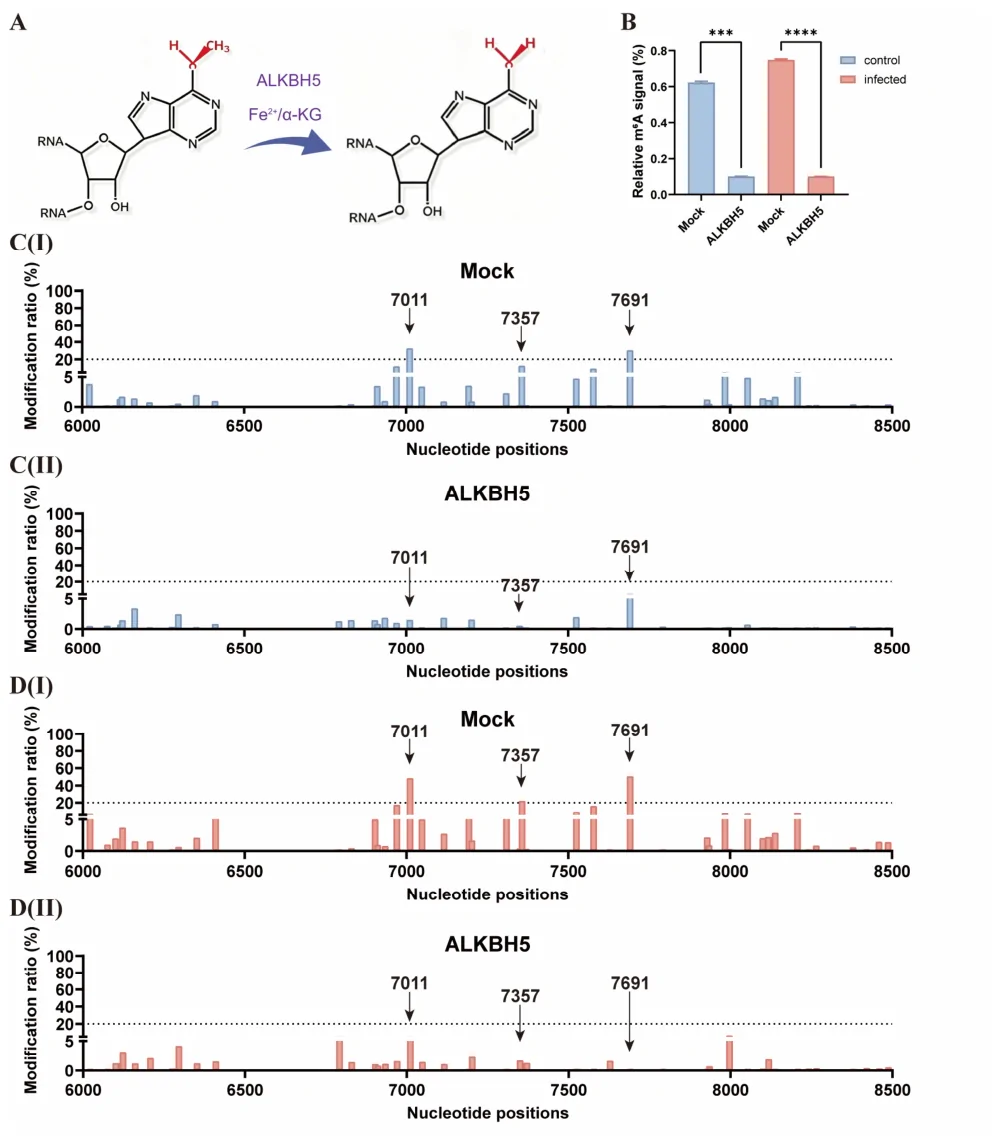
Effects of ALKBH5-mediated demethylation on global m^6^A levels and HTLV-1 m^6^A modification sites. (**A**) Schematic representation of ALKBH5-mediated m^6^A demethylation. (**B**) Relative global m^6^A levels in total RNA from control and HIV-1-infected MT-2 cells following mock or ALKBH5 treatment. (**C**) Modification ratios at HTLV-1 m^6^A sites in control MT-2 cells following mock or ALKBH5 treatment. (**D**) Modification ratios at HTLV-1 m^6^A sites in HIV-1-infected MT-2 cells following mock or ALKBH5 treatment. *** *p* < 0.001; **** *p* < 0.0001.

We further examined m^6^A modifications using ALKBH5-mediated demethylation (Figure 5A). In mock-treated samples, global m^6^A levels in total RNA showed a modest increase after HIV-1 infection, although the difference was not statistically significant (*p* > 0.05). ALKBH5 treatment significantly reduced global m^6^A levels in both groups (Figure 5B). Importantly, signals at HTLV-1 nucleotides 7011, 7357, and 7691 were strongly attenuated after ALKBH5 treatment (Figure 5C,D), supporting their assignment as m^6^A modification sites.

## 4. Discussion

In this study, we systematically characterized the effects of HIV-1 infection on the post-transcriptional regulation of HTLV-1 RNA in MT-2 cells. A central finding was that HIV-1 infection was associated with changes in HTLV-1 RNA processing without significantly changing total HTLV-1 RNA levels. The major changes involved alternative splicing, transcript composition, poly(A)-tail length, and RNA modification profiles. Thus, total HTLV-1 RNA levels alone do not fully reflect the effects of HIV-1 on HTLV-1 gene expression. Instead, our results show that HIV-1 infection is associated with changes in the processing and molecular properties of individual HTLV-1 RNA species. Previous studies reported increased HTLV-1/2 tax/rex transcripts or viral antigen expression during HIV-1 coinfection, whereas our findings extend these observations by defining the transcript-resolved post-transcriptional changes associated with this interaction [9,10,11,12].

Alternative splicing showed the most prominent changes following HIV-1 infection. D1-A8 splicing became predominant, accompanied by an increase in the proportion of p21^rex^ transcripts from 29.45% to 82.07% of the total HTLV-1 transcript population. At the same time, several other transcript classes became less represented, several low-abundance isoforms were no longer detected, and splice-site usage became less diverse. These coordinated changes indicate that HIV-1 infection does not simply alter a single HTLV-1 transcript, but redirects viral RNA processing toward a more restricted splicing program dominated by D1-A8 and p21^rex^. HTLV-1 generates multiple coding and regulatory RNAs through complex alternative splicing, and their expression is further shaped by Rex-dependent post-transcriptional regulation [13,14,15,16,21,22,23,25]. The predominance of p21^rex^ should be viewed as part of this broader redistribution of the HTLV-1 transcriptome. Its enrichment occurred together with reduced representation of env, gag-pro-pol, and several unannotated transcript classes. Because transcript abundance was calculated as a proportion of total HTLV-1 transcripts, the increase in the p21^rex^ fraction may reflect increased p21^rex^ production, reduced abundance of competing isoforms, or both. Absolute isoform-specific quantification will be required to distinguish these possibilities. Nevertheless, the magnitude of the shift from 29.45% to 82.07% demonstrates a redistribution of HTLV-1 transcript composition. This redistribution may alter the relative output of structural, regulatory, and accessory viral proteins and thereby influence subsequent stages of the HTLV-1 life cycle [26,27].

Changes in poly(A)-tail length showed that the effects of HIV-1 infection extended beyond alternative splicing. Importantly, these changes were transcript-specific and bidirectional. Env transcripts showed significantly longer poly(A) tails following HIV-1 infection, whereas p21^rex^, HBZ, and unannotated transcripts exhibited significant poly(A)-tail shortening; gag-pro-pol, tax/p27^rex^, and p13 remained unchanged. This pattern argues against a uniform effect on polyadenylation and instead indicates differential regulation of individual HTLV-1 RNA populations. Notably, env became less represented despite poly(A)-tail lengthening, whereas p21^rex^ became predominant despite poly(A)-tail shortening. Thus, transcript representation and poly(A)-tail state are not directly coupled in this setting, consistent with evidence that highly represented transcripts do not necessarily carry longer poly(A) tails [28,29,30].

Changes in m^6^A, m^5^C, and Ψ modification profiles of HTLV-1 RNA were also observed following HIV-1 infection. Nine additional modification sites were detected after infection, while modification levels at many sites present under both conditions also increased. The most extensive changes involved m^5^C and Ψ, whereas m^6^A showed comparatively more limited changes. At the transcript level, env, tax/p27^rex^, and defective-provirus-derived RNAs exhibited the strongest alterations, while gag-pro-pol remained comparatively stable. These findings indicate that HIV-1 induces selective and transcript-dependent changes in HTLV-1 RNA modification rather than a uniform shift across the viral transcriptome. Alternative splicing may contribute to part of this pattern by determining whether specific modification sites are retained in mature RNA molecules. ALKBH5-mediated demethylation further supported the assignment of HTLV-1 nucleotides 7011, 7357, and 7691 as m^6^A modification sites.

Taken together, these results indicate that HIV-1 does not exert a uniform effect on HTLV-1 RNA, but differentially affects multiple post-transcriptional processes across transcript classes. Several mechanisms may contribute to these changes. HIV-1 infection may alter the host RNA-processing environment in which HTLV-1 transcripts are spliced, exported, polyadenylated, and modified [62,63,64,65,66,67,68,69]. Changes in cellular splicing factors, RNA-binding proteins, 3′-end-processing factors, RNA-modifying enzymes, or RNA degradation pathways could collectively contribute to the transcript-specific patterns observed here. Shared nuclear-export machinery provides another plausible point of interaction, because HIV-1 Rev and HTLV-1 Rex both depend on CRM1-mediated export of incompletely spliced viral RNAs [70,71,72,73,74,75]. Together, these observations suggest that HIV-1 affects HTLV-1 RNA through multiple interconnected post-transcriptional processes rather than through a single regulatory pathway.

Several limitations of this study should be acknowledged. First, the present analysis was performed at a single time point, 36 h after HIV-1 infection. Therefore, the observed changes represent the post-transcriptional state of HTLV-1 RNA at this specific stage of infection and should not be assumed to remain unchanged at later time points. HIV-1-induced alterations in the cellular RNA-processing environment may evolve over time, and the magnitude or direction of changes in HTLV-1 splicing, poly(A)-tail length, and RNA modification may consequently differ during prolonged infection. Future time-course studies incorporating multiple post-infection time points will be required to determine the temporal dynamics and persistence of these changes.

Collectively, our data demonstrate that HIV-1 infection alters the post-transcriptional regulation of HTLV-1 RNA while leaving total HTLV-1 RNA levels essentially unchanged. These findings establish post-transcriptional regulation as an important layer of HIV-1/HTLV-1 interaction and provide a basis for further dissecting the mechanisms controlling HTLV-1 gene expression during HIV-1 coinfection.

## Data Availability

Raw Nanopore direct RNA sequencing reads have been deposited in the NCBI Sequence Read Archive (SRA). Data under BioProject PRJNA1513821 include the in vitro-transcribed HTLV-1 RNA standard (SRR40201624), two control MT-2 replicates (SRR40201625 and SRR40201626), which were previously reported by Wei et al. [44], and two HIV-1-infected MT-2 replicates (SRR40201627 and SRR40201628). For the ALKBH5-treated samples, sequencing data are available under BioProject PRJNA1513192, including the control MT-2 sample (SRR40187501) and the HIV-1-infected MT-2 sample (SRR40187500).

## Supplementary Materials

The following supporting information can be downloaded at: https://www.mdpi.com/article/10.3390/microorganisms14102359/s1, Figure S1: HIV-1 RNA levels in two independent HIV-1 infection experiments; Figure S2: Nanopore direct RNA sequencing characteristics of the second independent biological replicate; Table S1: Primer sequences for HTLV-1 in vitro transcription.

## Abbreviations

The following abbreviations are used in this manuscript:

HIV-1	human immunodeficiency virus type 1
HTLV-1	human T-lymphotropic virus type 1
RT-qPCR	reverse transcription quantitative PCR
DRS	direct RNA sequencing
TCID_50_	50% tissue culture infectious dose
MOI	multiplicity of infection
m^6^A	N^6^-methyladenosine
m^5^C	5-methylcytidine
Ψ	pseudouridine
ALKBH5	alpha-ketoglutarate-dependent dioxygenase AlkB homolog 5
