# Supplementary material for: HIV-1 Infection Alters Post-Transcriptional Processing of HTLV-1 RNA in MT-2 Cells
Source: Microorganisms. 2026 Oct 15;14(10):2359. doi: 10.3390/microorganisms14102359 (PMC13707466; doi:10.3390/microorganisms14102359)
Supplement: Supplementary file 1 [file microorganisms-14-02359-s001.zip › microorganisms-4602881_Supplementary_Materials.pdf]

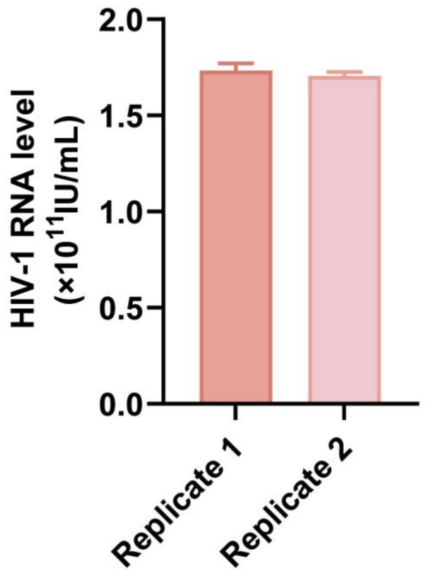

Figure S1. HIV-1 RNA levels in two independent HIV-1 infection experiments. MT-2 cells were infected with HIV-1 NL4-3 at an MOI of 0.5 and collected at 36 h post-infection. HIV-1 RNA levels in total RNA extracted from infected cells were quantified using an HIV-1 RNA Quantitative Diagnostic Kit. Replicate 1 and Replicate 2 represent two independent biological replicates.

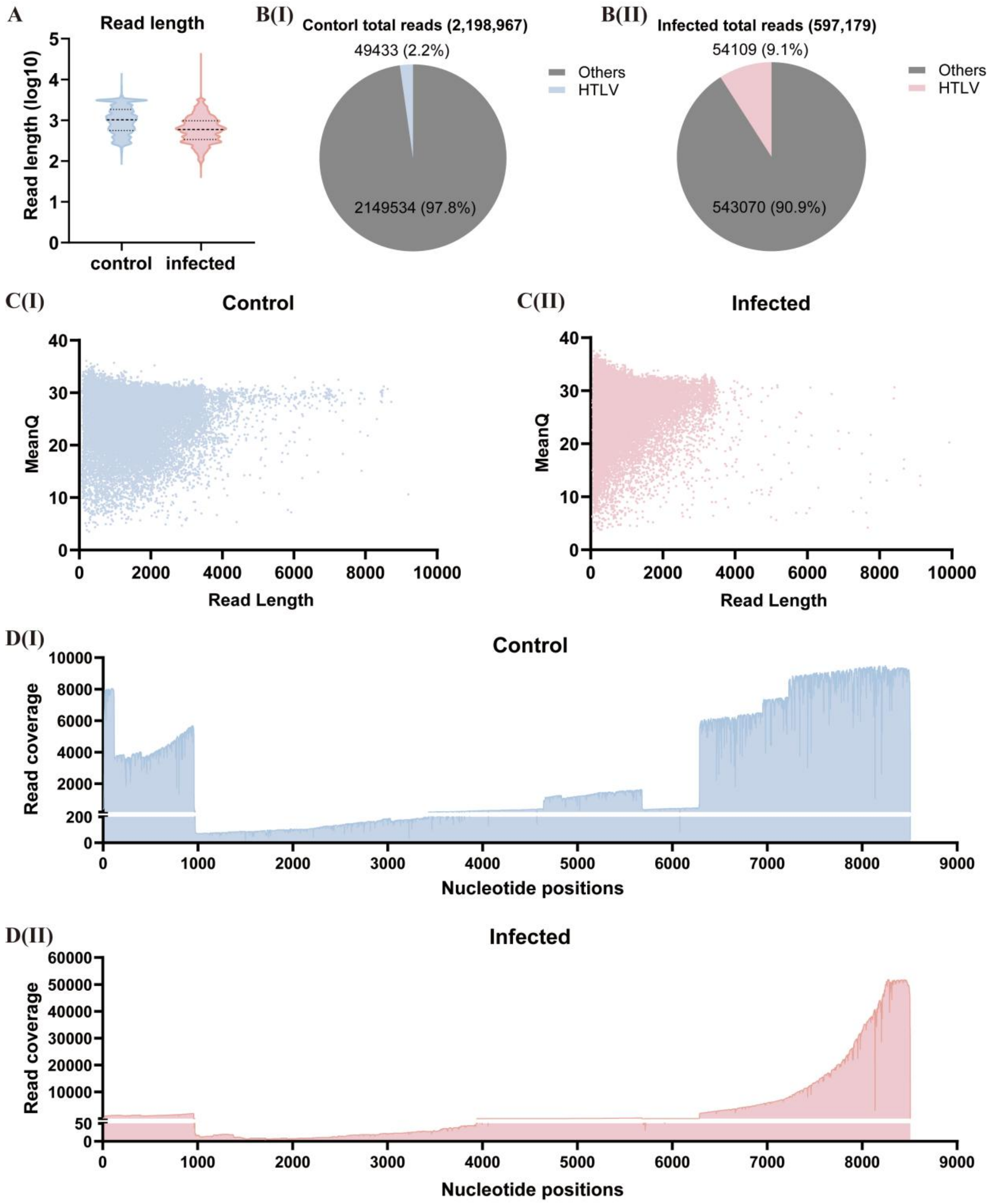

Figure S2. Nanopore direct RNA sequencing characteristics of the second independent biological replicate. (**A**) Log10-transformed read-length distributions of HTLV-1-mapped reads in control and HIV-1-infected MT-2 cells. (**B**) Numbers and proportions of HTLV-1 and non-HTLV-1 reads among the total sequencing reads in the two groups. (**C**) Scatter plots showing the relationship between read length and mean Q score in the two groups. (**D**) Read coverage across the HTLV-1 reference genome in the two groups. The control MT-2 direct RNA sequencing dataset shown in this figure was previously reported by Wei et al. [44] and is presented here for comparison with the corresponding HIV-1-infected dataset.

Table S1. Primer sequences for HTLV-1 in vitro transcription

Primer name	Primer sequence (5’→3’)
HTLV-IVT-1-F	CCCCTAATACGACTCACTATAGGGGGCTCGCATCTCTCCTTCAC
HTLV-IVT-1-R	TTTTTTTTTTTTTTTTTTTTTTTTTTTGGGTGTGGGATGTCG
HTLV-IVT-2-F	CCCCTAATACGACTCACTATAGGGAACCAGAGCCAGAGGAGGAT
HTLV-IVT-2-R	TTTTTTTTTTTTTTTTTTTTTTGGGCATGTAGCCAGACAAGT
HTLV-IVT-3-F	CCCCTAATACGACTCACTATAGGGCTATTATGCTGACCCTCACTGG
HTLV-IVT-3-R	TTTTTTTTTTTTTTTTTTTTTTGCTTCTTGAAGAGCCTCCTG
HTLV-IVT-4-F	CCCCTAATACGACTCACTATAGGGCCCTCAGCAATAAACAAACC
HTLV-IVT-4-R	TTTTTTTTTTTTTTTTTTTTTTCAAGGATAACAAGAAGGAGTAGC
HTLV-IVT-5-F	CCCCTAATACGACTCACTATAGGGAAGAGAGACCCCCCCTTGAA
HTLV-IVT-5-R	TTTTTTTTTTTTTTTTTTTTTATAGGCGTGCCATCGGTAAA
HTLV-IVT-6-F	CCCCTAATACGACTCACTATAGGGTTCCCTACAAACGAATAGAAAA ACT
HTLV-IVT-6-R	TTTTTTTTTTTTTTTTTTTTTAAGAGTTGAGCAAGCAGGGTC
